# Legendre Polynomial Fitting-Based Permutation Entropy Offers New Insights into the Influence of Fatigue on Surface Electromyography (sEMG) Signal Complexity

**DOI:** 10.3390/e26100831

**Published:** 2024-09-30

**Authors:** Meryem Jabloun, Olivier Buttelli, Philippe Ravier

**Affiliations:** Laboratoire Pluridisciplinaire de Recherche en Ingénierie des Systèmes, Mécanique, Énergétique (PRISME), University of Orleans, 45100 Orleans, France

**Keywords:** time series, multiscale permutation entropy, Legendre polynomial modelling, surface electromyography, fatigue

## Abstract

In a recently published work, we introduced local Legendre polynomial fitting-based permutation entropy (LPPE) as a new complexity measure for quantifying disorder or randomness in time series. LPPE benefits from the ordinal pattern (OP) concept and incorporates a natural, aliasing-free multiscaling effect by design. The current work extends our previous study by investigating LPPE’s capability to assess fatigue levels using both synthetic and real surface electromyography (sEMG) signals. Real sEMG signals were recorded during biceps brachii fatiguing exercise maintained at 70% of maximal voluntary contraction (MVC) until exhaustion and were divided into four consecutive temporal segments reflecting sequential stages of exhaustion. As fatigue levels rise, LPPE values can increase or decrease significantly depending on the selection of embedding dimensions. Our analysis reveals two key insights. First, using LPPE with limited embedding dimensions shows consistency with the literature. Specifically, fatigue induces a decrease in sEMG complexity measures. This observation is supported by a comparison with the existing multiscale permutation entropy (MPE) variant, that is, the refined composite downsampling (rcDPE). Second, given a fixed OP length, higher embedding dimensions increase LPPE’s sensitivity to low-frequency components, which are notably present under fatigue conditions. Consequently, specific higher embedding dimensions appear to enhance the discrimination of fatigue levels. Thus, LPPE, as the only MPE variant that allows a practical exploration of higher embedding dimensions, offers a new perspective on fatigue’s impact on sEMG complexity, complementing existing MPE approaches.

## 1. Introduction

During the past decades, the requirements for careful monitoring and diagnosis of the condition of the human motor system have evolved significantly. Surface electromyography (sEMG) is a commonly used technique in clinical settings that offers valuable insight on the functioning of the neuromotor system, force control, fatigue condition, and the diagnosis of neuromuscular disorders [1]. sEMG signals are noninvasive measures of the electrical activity produced by muscles, acquired near the surface of the skin. The genesis of sEMG involves the activation of motor units (MUs), each composed of a motoneuron and its associated muscle fibres [2]. The activation of MUs, known as recruitment, is a crucial aspect of force control and fatigue condition [3,4]. The complex interactions and nonlinear dynamics among various MU action potentials (MUAPs) contribute to both stochastic and deterministic components of the resulting sEMG signals [5,6].

Several entropy-based approaches have been explored to study fatigue conditions through the analysis of sEMG signals [6,7]. Among these, ordinal pattern (OP)-based entropy measures have emerged as promising tools for fatigue study. These measures have attracted attention due to their ability to efficiently transform a time series into a sequence of symbols, called OPs. This transformation relies on a unique parameter, a given embedding dimension *d*, which limits the symbol alphabet to a finite set [8,9,10,11,12]. Each symbol is derived through a threshold-free operation consisting of ranking each *d* successive samples in ascending order, guaranteeing robustness to linear perturbations and moderate noise levels in the obtained sequence [8,9]. The calculation of the occurrence frequency of OPs serves as an estimate of their probability distribution. The application of Shannon entropy to this OP probability distribution, referred to as permutation entropy (PE) in [8], provides insights into the temporal structure of the time series. In line with fatigue studies in [6,7], PE and its multiscale variants contribute to the widely accepted observation of fatigue–complexity correlations, where higher levels of fatigue are accompanied by a loss of complexity in sEMG signals. It is worth noting that conventional OP-based entropy measures [7,8,11,13,14] are unable to practically explore high embedding dimensions *d* to construct OPs due to a finite number of data samples. Indeed, the sample size needs to be significantly greater than d! to ensure accurate estimation of the OP probability distribution. In practice, *d* varies from three to seven [8,9]. As a result, the exploration of *d* samples at a given sampling frequency represents a significantly shorter duration compared to the time scale of physiological phenomena related to fatigue. This limits the comprehensive examination of the fatigue–sEMG complexity correlations.

In a previously published work [15], we introduced a multiscale PE (MPE) variant called Legendre polynomial fitting-based PE (LPPE). This LPPE enables the exploration of high embedding dimensions and employs local polynomial modelling of time series data to build OPs. By applying LPPE to various simulated datasets, we demonstrated its capacity to overcome the limitations of existing MPE variants, including sensitivity to noise and sampling frequency [15]. The current paper extends the work presented in [15] by applying LPPE to sEMG signals recorded during a fatiguing exercise. A comparison with the existing MPE variant, refined composite downsampling (rcDPE), is also provided. For a limited embedding dimension *d*, we observe that the LPPE value shows a systematic decrease as the fatigue level increases, consistent with the literature. However, the exploration of higher embedding dimensions in the current study unveils a different interpretation of the fatigue–sEMG complexity interaction, thereby better enhancing the differentiation of fatigue levels.

This paper is organised as follows: Section 2 briefly revisits the theoretical background of PE and LPPE. Section 3 details the synthetic and real sEMG data considered. Section 4 elaborates on the results, shedding light on the interpretation of LPPE under fatigue conditions. Finally, Section 5 concludes this paper and offers insights into ongoing research.

## 2. OP-Based Entropy Measures: A Brief Overview

This section presents the concept of OP and provides a brief overview of existing MPE variants, highlighting their limitations. It then describes how the LPPE proposed in [15] surpasses these limitations.

### 2.1. OP Concept

Let us consider *d* adjacent values $x_{t},x_{t+1},\ldots ,x_{t+d-1}$ obtained from a weakly stationary discrete-time sequence. By assuming rare occurrences of ties, where ties refer to equal values within the *d* considered samples, a unique OP, corresponding to the permutation allowing the ranking of these values in ascending order, can be obtained. The total number of distinct OPs is therefore d!. For example, the unique OP ‘24513’ of length d=5 is assigned to the five consecutive samples 1.1, 2.3, 3.4, −0.3, 1.2, while two consecutive OPs ‘2341’ and ‘3412’ of length d=4 are assigned to these same samples. Similarly, for d=3, these samples are coded by three consecutive OPs ‘123’, ‘231’, and ‘312’.

Accurately estimating the occurrence of an OP of type $\Pi_{i}$ and of order *d* requires a large sample number *N*, significantly surpassing the value of d!. This estimation can be achieved through the following expression:

(1) $$p_{\Pi_{i}}=\left\{\frac{\#t\;\;|\left\{x_{t},x_{t+1},\ldots ,x_{t+d-1}\right\}\;\mathrm{of}\;\mathrm{type}\;\Pi_{i}}{N-d+1}\right\},$$

where # denotes the cardinal. The OP concept presents an intuitive threshold-free transformation of a time series into a sequence of symbols. However, this transformation overlooks the growth rate of the signal and may assign the same symbol to sequences with different growth rates, such as for samples 0.1, 0.2, 0.3 and 0.001, 0.002, 0.003, both coded as OP 123 [16].

### 2.2. Limitation of Existing OP-Based Entropy Measures

Various OP-based entropy methods have been proposed in the literature [7,13,14,17,18,19]. The well-known method PE was introduced in [8] and is defined as the Shannon entropy applied to the probability distribution of OPs (1). Additionally, there exist variants of PE such as Renyi and Tsallis PE [20,21]. Another category of PE variants, known as MPEs, was designed to uncover multiscale signal structures. MPEs involve different estimation strategies of PE combined with linear preprocessing techniques, such as delay operators, linear filtering, and subsampling [7,13,18,22,23,24,25,26]. Alternatively, some MPEs combine PE estimators with nonlinear preprocessing techniques, such as data-driven signal decomposition [27,28,29,30,31,32,33,34,35,36,37,38,39,40,41,42,43].

The main limitations of PE and MPEs can be outlined as follows:

- Both PE and MPEs exhibit sensitivity to the chosen embedding dimension *d*. OPs of lower-order *d* may not adequately capture the complexity of the signal structure, resulting in a truncated interpretation of the results. Conversely, a higher-order *d* increases the PE computational cost and requires huge sample sizes to reduce bias and variance in the estimators of PE and MPEs [44].
- Additionally, both PE and MPEs are susceptible to noise and the sampling frequency [45,46]. Minor levels of noise can have a significant influence on the probability distribution of OPs at higher sampling frequencies. The sampling frequency itself can substantially modify the probability distribution of OPs, particularly when nearing the Nyquist rate.
- Moreover, MPEs relying on linear preprocessing are susceptible to aliasing artefacts induced by subsampling, particularly at larger scales [45,46,47].
- Another limitation is the insensitivity of PE and MPE to the growth rate of the signal.

Recent advancements have introduced alternative methods such as amplitude-aware PE [16,48], ensemble improved PE [49] and phase PE [17,50], which address some of the PE and MPE limitations by considering the growth rate, amplitude information, and phase patterns of the signal, respectively. Nonetheless, these approaches are also noise-sensitive. The ongoing challenges of developing robust OP-based entropy measures for analysing complex signals remains a significant concern.

In the following, before detailing LPPE, a robust OP-based entropy measure proposed in [15], we will briefly describe rcDPE, a variant of MPEs [7], primarily for comparison in the context of fatigue. The high sensitivity of rcDPE to fatigue conditions, as demonstrated in [7], sets it apart from classical MPEs.

### 2.3. rcDPE Measure

The rcDPE, as detailed in [7], computes the Shannon entropy of a single probability distribution of OPs through the following steps. Given a scale *M*, a maximum of *M* delayed versions of the raw signal are generated using linear lag operators τ=1, 2, …, M−1. Each delayed version is subsequently downsampled by a factor of *M* to form a composite signal. The probability of each OP is then estimated by averaging its occurrence across the set of the composite signals.

The rcDPE reduces artefact cross-correlation by alleviating redundancy among composite signal samples, a common issue in classical MPEs [7]. When applied to sEMG data collected during fatigue exercise, it exhibits superior performance in quantifying fatigue compared to classical MPEs [7]. These same sEMG data will be employed in the current study and discussed in more detail later.

### 2.4. LPPE Measure

In [15], a new variant of MPE, called the local Legendre polynomial modelling-based PE (LPPE), was introduced. This method involves modelling the signal locally using an orthonormal polynomial basis with a limited degree of d−1. First, the signal is divided into short segments of fixed length *L*, where L≥d. The choice of *L* value should satisfy two conditions: it must ensure local stationarity and allow for accurate polynomial modelling with a degree of d−1. The polynomial approximation of the signal, on each segment *i*, can be expressed according to Weierstrass’s theorem [51], as follows:

(2) $$x_{t}=\sum_{n=0}^{d-1}a_{i,n}P_{n}\left(t-i\right)+\epsilon_{t}\;\mathrm{for}\;t=i,i+1,\;\ldots ,\;i+L-1,$$

where $P_{n}\left(t\right)$ represents a polynomial of degree *n*, and $\epsilon_{t}$ denotes the approximation error. The well-known Legendre polynomials are used to construct the orthonormal discrete polynomial basis, $P_{n}\left(t\right)$ with n=0, 1, …, d−1.

For each segment *i*, the *d* model parameters, denoted by $a_{i,n}$, are estimated using a local least squares (LS) strategy:

(3) $${\hat{a}}_{i,0},\;{\hat{a}}_{i,1},\;\ldots ,\;{\hat{a}}_{i,d-1}=\underset{a_{i,0},a_{i,1},\;\ldots ,\;a_{i,d-1}}{\arg\min}\sum_{t=i}^{i+L-1}{\left|x_{t}-\sum_{n=0}^{d-1}a_{i,n}P_{n}\left(t-i\right)\right|}^{2}\;\;.$$

The obtained parameters (3) are then ranked to generate an OP of length *d* [15]. Once all segments have been processed, the occurrence of an OP of type $\Pi_{i}$ and of order *d* can be determined using the following formula:

(4) $$p_{\Pi_{i}}=\left\{\frac{\#t\;\;|\left\{{\hat{a}}_{t,0},\;{\hat{a}}_{t,1},\;\ldots ,\;{\hat{a}}_{t,d-1}\right\}\mathrm{of}\;\mathrm{type}\;\Pi_{i}}{N-L+1}\right\}.$$

Finally, the normalised Shannon entropy is computed using the resulting OP probability distribution [15]:

(5) $$H=-\frac{\sum_{i}p_{\Pi_{i}}\log\left(p_{\Pi_{i}}\right)}{\log(d!)}.$$

It is important to highlight that by adjusting *L* and *d* under the constraint L≪N and d≤L, we explore the multiscale aspect of LPPE. Unlike classical MPEs, which rely on limited sample sizes to generate OPs, LPPE allows for the exploration of a larger number of samples to produce OPs of length *d*. This not only enhances the characterisation of signal complexity but also helps avoid the risk of truncated interpretation in PE analysis.

Moreover, as demonstrated in [15], LPPE exhibits increased robustness to fluctuations and noise. This is attributed to the orthonormality property of the polynomial basis, ensuring uncoupled parameters with similar value ranges and enabling a reliable ranking procedure. The obtained model parameters (2) implicitly provide information about the first *d* higher-order derivatives of the signal, such as local growth rate (first derivative), local convexity/concavity (second derivative), and so forth. This contributes to a more comprehensive interpretation of the PE-based complexity measure. Additionally, LPPE does not require subsampling, thereby avoiding aliasing problems.

## 3. Methods

To ascertain the sensitivity and ability of the LPPE method in extracting relevant information from sEMG signals for fatigue study, we consider both synthetic and real sEMG data. Firstly, we recall the basic model used for generating synthetic sEMG signals [52]. Then, we provide a brief overview of the experimental setup for data collection and the requisite data preprocessing steps prior to the application of PE-based techniques. Readers are encouraged to refer to [7,53,54] for more details.

### 3.1. Simulated Data

The model proposed by Shwedyk et al. [52] for simulating sEMG signals relies on the generation of filtered white Gaussian noise characterised by a known power spectral density (PSD). This PSD expression is given by the following [52]:

(6) $$PSD\left(f\right)\propto \frac{f_{h}^{4}f^{2}}{\left(f^{2}+f_{l}^{2}\right){\left(f^{2}+f_{h}^{2}\right)}^{2}},$$

where its shape can vary thanks to two frequency parameters, $f_{l}$ and $f_{h}$. In our study, four parameter pairs are considered, denoted as $w_{1}$ to $w_{4}$:

(7) $$\begin{array}{ccccc} \left(f_{l},f_{h}\right) & w_{1} & w_{2} & w_{3} & w_{4}\\ f_{l}\;\left(\mathrm{Hz}\right) & 49 & 49 & 39 & 29\\ f_{h}\;\left(\mathrm{Hz}\right) & 146.5 & 117 & 98 & 58.5 \end{array}$$

**Figure 1:**
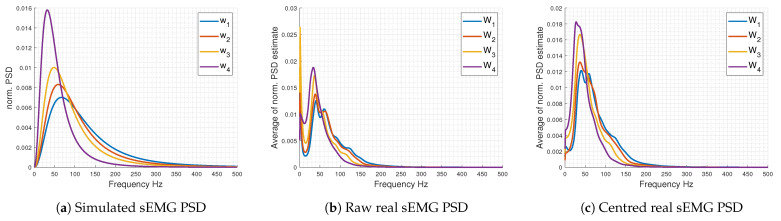
Normalised sEMG PSDs: (**a**) Simulated PSDs calculated using (6) and parameter pairs $\left(f_{l};f_{h}\right)$ in Hz: (49;146.5) denoted as $w_{1}$, (49;117) as $w_{2}$, (39;98) as $w_{3}$, and (29;58.5) as $w_{4}$; 50 Monte Carlo realisations of synthetic sEMG signals based on filtered white Gaussian noise are generated for each parameter pair. (**b**) Average normalised PSD estimates of sEMG signals acquired under fatigue conditions $W_{1}$ to $W_{4}$, as described in Section 3.2, and subsampled by a factor of 10. (**c**) Average normalised PSD estimates of these same sEMG signals after mean removal. All PSD estimates were calculated using an AR model of order 30 [55].

A total of 50 realisations of synthetic sEMG are performed for each parameter pair. The sampling frequency $F_{s}$ is fixed at 1000 Hz, and the sample number is 10,000. Figure 1a displays the expected synthetic sEMG PSDs for $w_{1}$ to $w_{4}$, which closely resemble the PSDs of real sEMG signals acquired, as described in the subsequent subsection.

### 3.2. Experimental and Data Setup

Data for this study were obtained from a previous experiment detailed in [7,53,54], where they were investigated using different indicators, namely, fractal indicators, spectral parameters, and the complexity measure rcDPE. This experiment involved ten healthy participants (three females and seven males, aged 24 ± 1.5 years, all right-handed, briefed on the experimental protocols; each provided written consent). Here, we provide an outline of the experiment conducted according to the guidelines of the “Declaration of Helsinki”, and approved by the Institutional Pedagogical and Ethical Committee of “Institut National de Formation Supérieure en Sciences et Technologies du Sport (INFS/STS) de Dely Ibrahim, Alger”, Algeria (2004).

Participants were seated and securely fastened at the waist and shoulders, with the right arm horizontally positioned and supported. An isometric ergometer was custom-designed for the experiment to facilitate standardised isometric flexion contractions of the elbow. The elbow joint was set at a 100-degree extension angle and monitored using an electronic goniometer (Alpha-C Kosmos digital meters A/D converter, Ditel, Saint-Etienne, France), with the hand in a neutral position. A wrist cuff connected to the strain gauge displayed force levels on a screen to the subject using a cursor. The force levels for the fatigue test were set using a visual target. Force measurements were captured using a strain gauge (0–2000 N range, ZF, Scaime, Haute-Savoie, France), conditioned to a 1000 Hz frequency bandwidth.

The sEMG signals were recorded from the short head of the biceps brachii using surface electrodes placed midway between the motor innervation point and the tendon after preparing the skin. These electrodes were manufactured by In VIVO METRIC (Ventura, CA, USA) and were of the two round bipolar silver–silver chloride (Ag-AgCl) type (E220N model, sensor 4 mm diameter, gel cavity 2 mm deep). These electrodes are compressed into 1 mm thick sensor disks connected to lead wire and firmly encapsulated into durable epoxy housing. The recorded sEMG signals were amplified using bipolar isolated amplifiers (common mode rejection ratio of 150 dB, 2–600 Hz band-pass filter) [56]. Both force and sEMG signal acquisitions were synchronised using an analogue-to-digital card (PCI 6023E, National Instrument, Austin, TX, USA) at a 10 kHz sampling frequency.

The experimental protocol comprised three stages of isometric elbow flexion contractions. The first stage involved a brief warm-up with low-intensity and short-duration contractions. In the second stage, participants performed three maximal contractions of less than 3 s each, separated by 3-min rest intervals, to determine the maximal voluntary contraction (MVC) and set the contraction intensity level for the fatigue test. In the final stage, participants performed the fatigue test, maintaining contractions at 70% MVC until exhaustion, defined as the point at which they could no longer sustain a constant force level.

For comparison purposes with the study presented in [7], each sEMG signal recorded from sustained force exertion until exhaustion is partitioned into four nonoverlapping segments of equal length, referred to as windows ($W_{1}$, $W_{2}$, $W_{3}$, and $W_{4}$). These windows represent distinct sequential stages of exhaustion (0–25%, 25–50%, 50–75%, and 75–100% of the time to exhaustion, respectively). Figure 1b,c display the average PSD estimates of the acquired sEMG signals subsampled by a factor 10 across the ten participants for each window $W_{i}$. We propose to analyse each window $W_{i}$ of the collected datasets using LPPE in comparison to rcDPE. As discussed in [7], the latter has demonstrated superior sensitivity to fatigue conditions compared to classical MPEs.

## 4. Results

This section is dedicated to the performance assessment of the LPPE when applied to both simulated sEMG signals and real sEMG signals acquired during fatigue exercise. A comparison with rcDPE is also provided. Both considered methods are calculated using *d* = 4 and 5. These values are chosen in accordance with the recommendations from previous studies [8,9,44,54]. Specifically, *d* should satisfy the constraint N≫d! to ensure accurate estimation of the OP probability distribution. Additionally, for rcDPE, *d* should satisfy $\frac{N}{M}\gg d$ to ensure low MPE bias [7]. Furthermore, in [54], it was found that *d* = 3 primarily reflects shifts in the signal spectrum. Therefore, it is beyond the scope of the current paper.

### 4.1. Simulated sEMG Processes

**Figure 2:**
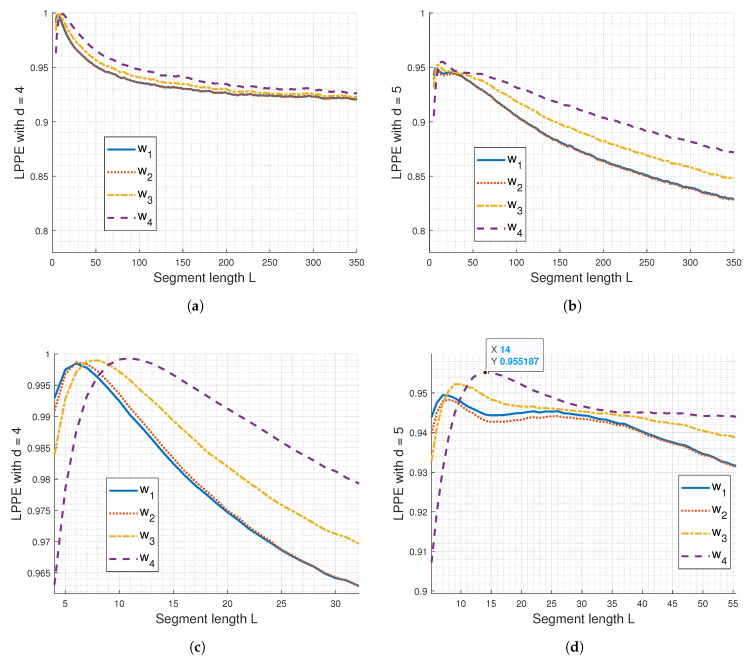
LPPE with (**a**) d=4 and (**b**) d=5, applied to synthetic sEMG signals generated as described in Section 3.1. (**c**,**d**) show a zoom performed on (**a**) and (**b**), respectively. The sampling frequency is 1000 Hz.

Figure 2a,b display the results obtained by applying the LPPE method with OP length d=4 and 5 to synthetic sEMG signals generated as described in Section 3.1. The embedding dimension, represented here by the segment length *L*, determines the number of samples considered to generate an OP. This segment length can be read in samples or straightforwardly converted to ‘Duration’ in milliseconds, since the sampling frequency is 1 kHz. No comparison with rcDPE is presented in this subsection as it is consistently affected by aliasing.

From Figure 2a,b, it is evident that the discrimination between LPPE curves for $w_{1}$, $w_{2}$, $w_{3}$, and $w_{4}$ is better enhanced using d=5. Additionally, the selection of *L* significantly impacts the LPPE measures, as shown in the zoom-in of Figure 2a,b, depicted in Figure 2c,d, respectively. Indeed, from these latter figures, we observe the presence of LPPE maxima and notice two main cases of LPPE behaviour regarding the *L* value in relation to these maxima:

- First case: When L≤6 ms for d=4 and L≤7 ms for d=5, the LPPE values for $w_{4}$ are the lowest, followed by those for $w_{3}$, $w_{2}$, and finally $w_{1}$, which has the highest LPPE. In this case, the Legendre polynomials, with a limited degree of *d*-1 and a small embedding dimension *L*, are more effective at capturing high-frequency content than low-frequency content of the simulated sEMG signals, which are broadband random signals. The probability distribution of the built OPs is spread across many possible OPs, indicating a high level of randomness. This spread increases as the high-frequency range of these simulated sEMG signals increases, leading to the observed LPPE behaviour from $w_{4}$ to $w_{1}$.
- Second case: When using L≥20 ms for d=4 and L≥35 ms for d=5, the highest LPPE values are observed for $w_{4}$, while the lowest LPPE values are seen for $w_{1}$ and $w_{2}$, which exhibit superimposed curves, regardless of the *d* value. In this scenario, the Legendre polynomials with a limited degree of *d*-1 and a high embedding dimension *L* are less effective at capturing the high-frequency content of the simulated sEMG signals. Instead, they primarily account for the low-frequency components, resulting in OPs that reveal randomness in these lower frequencies. As the embedding dimension increases, the probability distribution of OPs becomes more concentrated on a few highly probable OPs, leading to a lower LPPE measure. This concentration on fewer OPs becomes more pronounced as the low-frequency range of the simulated sEMG signals decreases, which explains the observed LPPE behaviour from $w_{4}$ to $w_{1}$.

In both cases, no relevant oscillations are noticed in the LPPE curves; however, they exhibit a decreasing trend that clearly reaches a plateau at d=4 for higher values of *L*.

The presence of LPPE maxima for both considered values of *d*, observed in Figure 2c,d, may reflect dominant frequencies captured by OPs of length *d* (see Appendix A for details). Table 1 lists the segment lengths *L* contributing to the LPPE maxima and their potentially associated main frequency bands detected by LPPE. For comparison, the median frequencies of simulated sEMG signals are also reported. These detected frequency bands align with the shift towards lower frequencies in simulated sEMG PSDs, previously illustrated in Figure 1a,c [55].

### 4.2. Real sEMG Signals

For comparison purposes, we first recall the results obtained using rcDPE applied to the real sEMG signals acquired under fatigue conditions [7]. We then present the LPPE results, emphasising their complementarity.

#### 4.2.1. rcDPE Results

Given that each recorded sEMG signal comprises approximately 274,000 samples, we continue to calculate the rcDPE using OP lengths limited to d=4 and d=5. Recall that these values also represent the embedding dimension for the rcDPE method. A range of scale values *M* from 1 to 10 in one-step increments was tested for rcDPE before encountering aliasing due to downsampling effects. It is worth noting that the particular scale M=10 results in a new sampling frequency of Fs=1000 Hz, which is known to be suitable for a moderate contraction of the biceps brachii muscles with an equivalent bandwidth of 500 Hz [7,53,55].

**Figure 3:**
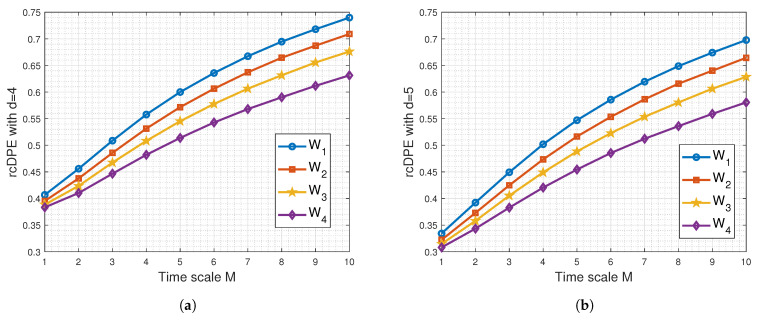
Mean rcDPE with (**a**) d=4 and (**b**) d=5 applied to real sEMG signals acquired as described in Section 3.2. The rcDPE is insensitive to the mean removal of the acquired sEMG signals.

As observed in Figure 3, the rcDPE averages calculated using the sEMG signals increase with the scale *M*. At any fixed scale, the rcDPE average is lowest for $W_{4}$, followed by $W_{3}$, then $W_{2}$, with the highest being $W_{1}$. This result is consistent with findings from classical MPEs and aligns with the widely accepted notion that fatigue induces lower sEMG signal complexity [6,7].

#### 4.2.2. LPPE Results

For the LPPE, the embedding dimension *L* can be much higher as long as it allows for an unbiased estimation of the probability distribution of built OPs. However, to ensure weak stationarity of the segments, we limit *L* to segments of 600 ms [55]. The observations detailed in what follows remain consistent regardless of any subsampling performed on the signals, provided that no aliasing occurs. To aid interpretation, we present results obtained from signals subsampled by a factor of M=10 and using an anti-aliasing filter, resulting in a new sampling frequency of $F_{s}=1000$ Hz. Therefore, *L* can be read either as a sample number or as a duration in milliseconds (ms).

**Figure 4:**
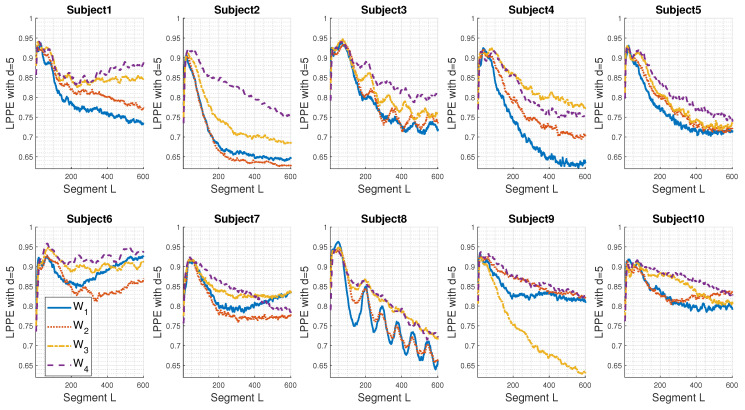
LPPE with d=5 applied to real sEMG signals, acquired under fatigue condition from 10 subjects as described in Section 3.2 and subsampled by a factor M=10. The sampling frequency is $F_{s}$ = 1000 Hz.

Figure 4 displays the individual LPPE measures of the real sEMG signals acquired under fatigue conditions ($W_{1}$ to $W_{4}$) for the ten subjects considered.

As can be noticed from Figure 4, individuals present different LPPE shapes with respect to the segment length *L*. However, except for Subject 9, similar behaviours are observed for higher values of L∈[100; 300] ms: the LPPE for $W_{1}$ and $W_{2}$ are close and are the lowest values. On the other hand, the LPPE for $W_{3}$ and $W_{4}$ are also close, with $W_{4}$ usually exhibiting the highest values. Subject 2 shows the biggest differentiation between the four conditions $W_{1}$ to $W_{4}$, with a steady decrease in the LPPE. Additionally, we observe oscillations in LPPE values, such as those in Subject 8, which appear to be sinusoidal with a main period of approximately 100 ms.

**Figure 5:**
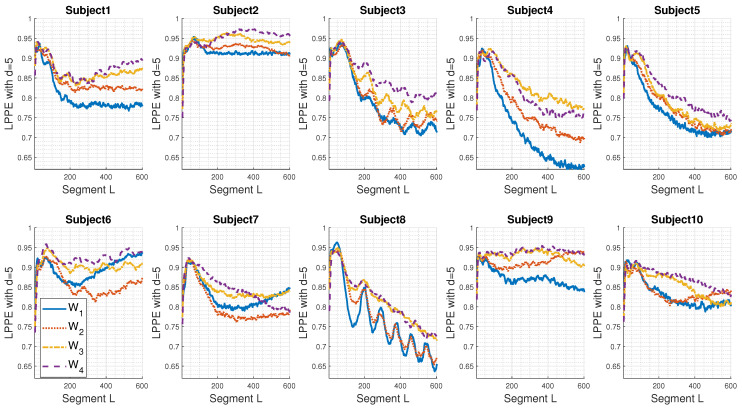
LPPE with *d* = 5 applied to centred (mean removal) real sEMG signals acquired under fatigue condition from 10 subjects as described in Section 3.2. The sampling frequency is $F_{s}$ = 1000 Hz.

Regarding Subject 9, the decrease in LPPE for $W_{3}$ (Figure 4) does not follow the pattern observed in other individuals. Nevertheless, a quick view of Figure 5, where LPPE values are calculated after the mean removal of the recorded sEMG signals, shows that the LPPE behaviour of Subject 9 is similar to that of the other subjects. Clearly, it seems that the continuous mean of the raw sEMG signal of Subject 9, i.e., an offset, has a significant impact. The LPPE has captured this as a dominant, very-low-frequency component for higher embedding dimensions (L>40).

We report in Table 2 the specific values $L_{Maxima}$ of *L* that contribute to the maximum LPPE, calculated with *d* = 5, and for the 10 considered subjects. It is evident that these values are far from those observed in the studied synthetic sEMG signals. However, we still notice a consistent shift towards lower frequencies as the fatigue level increases.

**Figure 6:**
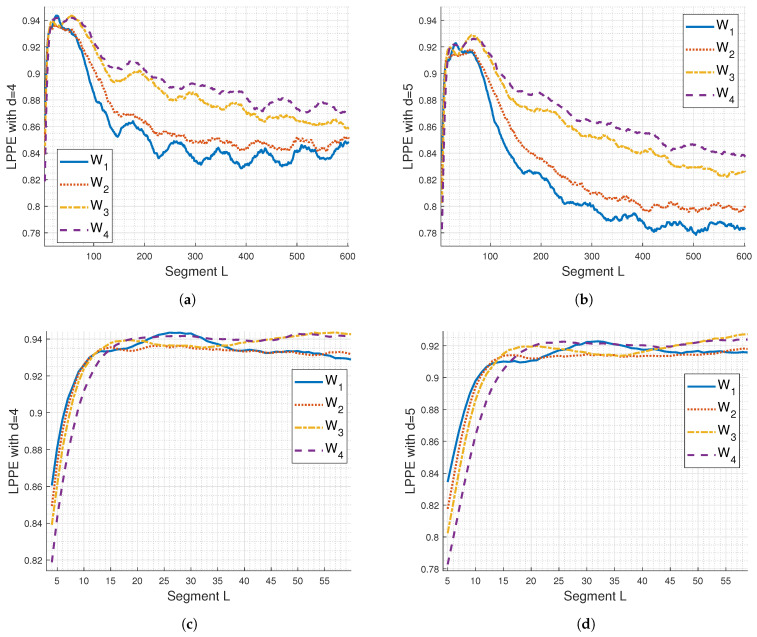
Mean LPPE with (**a**) *d* = 4 and (**b**) *d* = 5 using the 10 real sEMG signals acquired as described in Section 3.2, after mean removal and subsampling by *M* = 10. (**c**,**d**) are a zoom of (**a**) and (**b**), respectively. The sampling frequency is $F_{s}$ = 1000 Hz.

To further the analysis, Figure 6 displays the LPPE averages calculated from the sEMG signals of the 10 subjects for both d=4 and d=5. It is evident that the differentiation between the four conditions ($W_{1}$ to $W_{4}$) is more pronounced using d=5 and segment lengths *L* ranging from 200 to 450 ms. We also observe LPPE maxima and two main cases of LPPE behaviour regarding *L* in relation to these maxima, similar to the simulated sEMG signals: the first case where L≤15 ms, and the second case where *L* corresponds to a duration longer than 75 ms. In the first case, it is notable that the LPPE results are consistent with those obtained using rcDPE (see Figure 3 and Figure 6c,d) or any conventional MPEs. The interpretation of LPPE behaviour provided in Section 4.1 remains applicable to both cases.

**Figure 7:**
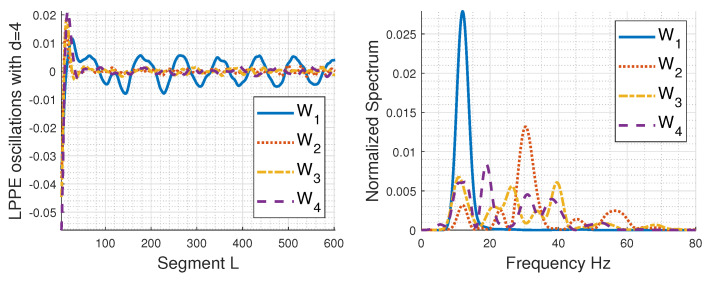
Oscillations in detrended LPPE of real sEMG signals obtained using the data-driven decomposition method, VMD, and their respective spectra with *d* = 4. The sampling frequency is $F_{s}$ = 1000 Hz.

However, a key difference from the simulated sEMG signals is that the LPPE averages of real sEMG signals exhibit higher oscillations, which should be highlighted. Figure 7 depicts these oscillations for *d* = 4 and their spectra after trend removal, using the effective data-driven method Variational Mode Decomposition (VMD) [57]. Similar results are obtained using *d* = 5. We notice the presence of dominant frequency bands in these oscillations:

- 9–20 Hz band: This band is present across all conditions but is highly pronounced in $W_{1}$ and lowest in $W_{2}$.
- For $W_{2}$, a pronounced peak is observed around 30 Hz.
- 20–40 Hz band: For $W_{3}$, there are two pronounced peaks around 25 Hz and 40 Hz within this band. For $W_{4}$, the spectrum energy is spread within this band, with one peak at 20 Hz, followed by less prominent peaks at 30 Hz and 40 Hz.

Future investigations should focus on whether these oscillations can potentially be linked to physiological parameters: the motor unit firing rate (MUFR), physiological tremor, fatigue-related tremor, velocity conduction, etc. In nonfatigued muscle activity, the MUFR typically ranges from 8 Hz to 20 Hz [55,58,59,60] but changes as muscle fatigue progresses. Physiological tremor occurs with a frequency range of 8–12 Hz, while the frequency range of fatigue-related tremor can be higher or lower depending on many factors [61,62,63]. However, exploring this task requires extensive research and is beyond the scope of the current paper.

#### 4.2.3. LPPE versus rcDPE

**Figure 8:**
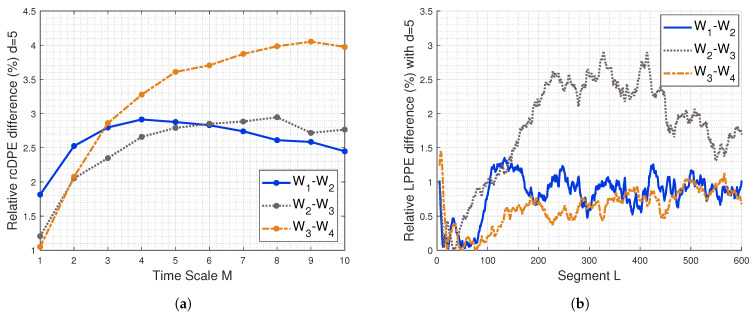
Relative absolute difference of (**a**) LPPEs and (**b**) rcDPE of real sEMG signals using pairwise comparisons of the fatigue steps $W_{1}$-$W_{2}$, $W_{2}$-$W_{3}$, and $W_{3}$-$W_{4}$. The sampling frequency is 1000 Hz. The relative absolute difference is calculated using $100\times \frac{|y_{i}-y_{j}|}{|y_{i}+y_{j}|}$, where $y_{i}$ is LPPE or rcDPE of step $W_{i}$.

To compare the sensitivity of LPPE and rcDPE to fatigue, the relative absolute differences between rcDPE evaluated under consecutive conditions are shown in Figure 8a, while those of LPPE are shown after mean removal of raw sEMG signals in Figure 8b.

From the LPPE results, we observe that the conditions $W_{2}$ and $W_{3}$ exhibit the highest differences. Conditions ($W_{1}$, $W_{2}$) and conditions ($W_{3}$, $W_{4}$) exhibit similar differences for an embedding dimension corresponding to a time duration higher than 200 ms. Notably, for low-value embedding dimensions, the LPPE results show tendencies similar to those of rcDPE, especially at *M* = 10. Both methods provide complementary information for distinguishing fatigue conditions.

Connections to known physiological factors can help explain the differences observed between the dynamics of rcDPE and LPPE as fatigue levels increase. As reported in [6,7,64,65,66,67], during sustained isometric exercises—such as the fatigue exercise examined in this study—progressive physiological changes associated with fatigue occur. Notably, studies have observed an increase in the duration of action potentials, along with a corresponding lengthening of motor unit action potentials (MUAPs) [65,67]. Since sEMG signals represent the summation of these MUAPs, this lengthening could increase the probability of ascending or descending OPs built from fewer consecutive samples, as is the case in rcDPE and LPPE with small *L*. This imbalance in the OP probability distribution leads to lower entropy values as fatigue progresses.

In contrast, LPPE can build free-aliasing OPs of length *d* using larger sample number *L*. These OPs reflect the overall trend of the high-frequency components that dominate the sEMG signal power at the beginning of muscle contraction, resulting in a more unbalanced OP distribution and thus lower entropy values. As fatigue progresses and low-frequency components become dominant, the OP distribution becomes less unbalanced, causing an increase in entropy values.

## 5. Conclusions

In this paper, we analysed sEMG signals acquired under fatigue conditions using two multiscale permutation entropy methods: the rcDPE and a novel variant of MPE called LPPE. Thanks to the practical and theoretical advantages of polynomial approximation to construct OPs using a larger amount of sample data, and being a free-aliasing multiscaling conception, LPPE achieved a more sensitive capture of the signal’s growth rate and derivatives often overlooked in traditional MPEs. Consequently, LPPE offered complementary insights into the impact of fatigue on sEMG complexity.

We observed that LPPE, when calculated with a high embedding dimension, better highlighted the transition between real sEMG signals acquired during sequential stages of exhaustion 25–50% and 50–75%. The comparison with an academic model used to simulate sEMG signals revealed that LPPE exhibited noticeable oscillations for real acquired sEMG signals, which were not significant in the synthetic ones. This suggests that the synthetic model may not fully capture the full structure of real sEMG signals. Future research will focus on using physiological models to better simulate sEMG signals under fatigue and to explore whether there is a potential link between their LPPE variations and motor unit firing rates.

## Figures and Tables

**Table 1:** LPPE applied to simulated sEMG signals reaches maximum for particular embedding dimension $L=L_{Maxima}$. The sampling frequency is 1000 Hz. The reported frequency bands are roughly estimated, based on the $L_{Maxima}$ value, using the approximate curve shown in Figure A1b, as explained in Appendix A.

	Median Frequency	d=4		d=5	
	(Hz)	$L_{\mathrm{Maxima}}$ (ms)	Freq. (Hz)	$L_{\mathrm{Maxima}}$ (ms)	Freq. (Hz)
$w_{1}$	101	6	82–95	7	72–81
$w_{2}$	87	6	82–95	8	64–71
$w_{3}$	72	8	64–71	10	53–57
$w_{4}$	47	11	48–51	14	39–41

**Table 2:** Segment length *L* corresponding to the LPPE maximum of individual sEMG signals acquired under the four fatigue conditions. The LPPE is calculated using *d* = 5, and the sampling frequency is $F_{s}$ = 1000 Hz.

Subject	$W_{1}$		$W_{2}$		$W_{3}$		$W_{4}$	
	$L_{Maxima}$	Freq.	$L_{Maxima}$	Freq.	$L_{Maxima}$	Freq.	$L_{Maxima}$	Freq.
1	28	20.4	11	51.9	13	44	16	35.7
2	19	30.1	21	27.2	25	22.9	30	19
3	58	9.9	71	8	63	9.1	64	8.9
4	30	19	28	20.4	70	8.2	25	22.8
5	20	28.6	16	35.7	16	35.7	17	33.6
6	61	9.4	64	8.9	59	9.7	59	9.7
7	29	19.7	34	16.8	35	16.3	37	15.4
8	43	13.3	54	10.6	43	13.3	35	16.3
9	15	38.1	14	40.8	24	23.8	24	23.8
10	23	24.8	16	35.7	55	10.4	72	7.9
Mean	32.6	21.3	32.9	25.6	40.3	19.3	37.9	19.3

**Figure A1:**
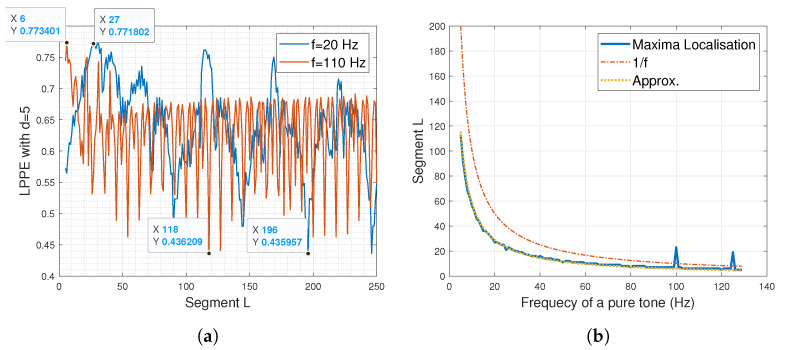
LPPE of pure tones: (**a**) LPPE shapes are shown for two sinusoids of frequencies 20 Hz and 110 Hz, with *d* = 5. (**b**) Segment length *L* corresponding to LPPE maxima represented as a function of the frequency of the pure tone. Two additional curves are provided: (-.) the inverse of the frequency, and (:) this curve scaled by a factor of 0.57 as an approximation. The sampling frequency is 1000 Hz.

**Figure A2:**
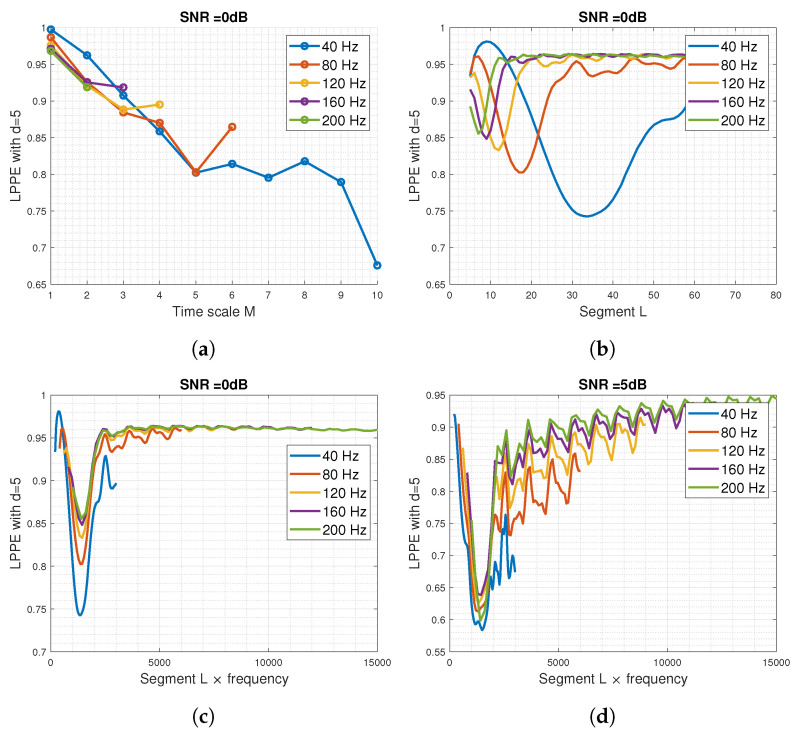
LPPE of sinusoids embedded in an additive Gaussian noise: (**a**) rcDPE and (**b**) LPPE with SNR = 0 dB. (**c**,**d**) are LPPE plotted as a function of embedding dimension × the sinusoid frequency at SNR 0 and 5 dB.

## Data Availability

Data contains in this article.

## Appendix A. LPPE of Pure Tones

To ensure this paper is self-contained, we provide additional information on LPPE applied to academic signals. For further examples with narrow- and wideband signals, readers are encouraged to refer to [15].

Figure A1a illustrates the LPPE with d=5 for two pure tones with distinct frequencies, 20 Hz and 110 Hz, sampled at $F_{s}$ = 1000 Hz. The number of samples corresponds to 1000 time periods. The oscillations in LPPE reflect the frequencies of the pure tones, with both sinusoids achieving their lowest LPPE values around 0.43 but for two different segment lengths *L*. Both curves exhibit LPPE maxima.

In Figure A1b, we present the segment lengths *L* corresponding to the LPPE maxima of pure sinusoids as a function of their frequencies, varying from 5 Hz to 130 Hz. Additionally, we provide two curves: one depicting the inverse of the frequency and another representing this curve scaled by a factor of 0.57 as a rough approximation. The sampling frequency remains 1000 Hz. Notably, as the frequency of the pure tone increases, the segment length *L* corresponding to the LPPE maximum decreases until it reaches the plateau limit L=d. Similar curves can be obtained with *d* = 4. Based on the curves in Figure A1b, given an *L* value, we can estimate the corresponding dominant frequency band as $f\in \left[\frac{0.57\;F_{s}}{L+1},\frac{0.57\;F_{s}}{L-1}\right]$.

## Appendix B. LPPE of Pure Tones Embedded in Noise

Figure A2a,b compare the LPPE and rcDPE for sinusoidal signals embedded in additive Gaussian noise (AWGN) with signal-to-noise ratios (SNRs) fixed at 0 dB. The oscillations in LPPE reflect the frequency of the sinusoids, as illustrated in Figure A2c,d for SNRs fixed at 0 and 5 dB. In other words, Figure A2b,c display the same information but in two different ways. In these latter figures, LPPE is plotted as a function of the segment length (L: embedding dimension) multiplied by the frequency of the sinusoids.
